# KC-like chemokine as a biomarker of sepsis in dogs with pyometra

**DOI:** 10.1186/s12917-024-04271-w

**Published:** 2024-09-13

**Authors:** Ragnvi Hagman, Caroline Klemming, Emma Bengtsdotter, Fredrik Södersten, Liya Wang, Sara Wernersson

**Affiliations:** 1 Department of Clinical Sciences, Swedish University of Agricultural Sciences, Box 7054, Uppsala, 75007 Sweden; 2 Department of Anatomy, Physiology and Biochemistry, Faculty of Veterinary Medicine and Animal Science, Swedish University of Agricultural Sciences, Box 7011, Uppsala, 75007 Sweden; 3 Department of Pathology and Wildlife diseases, National Veterinary Institute (SVA), Ulls väg 3, Uppsala, 75189 Sweden; 4 Department of Biomedical Science and Veterinary Public Health, Swedish University of Agricultural Sciences, Box 7028, Uppsala, 75007 Sweden

**Keywords:** Canine, Pyometra, Sepsis, SIRS, KC-like, CXCL1

## Abstract

**Background:**

Sepsis, defined as a dysregulated inflammatory response to infection inducing organ dysfunction, is a common cause of mortality in both humans and animals. Early detection and treatment is essential for survival, but accurate diagnosis is challenging due to the lack of specific biomarkers for sepsis. This study explored the potential of the keratinocyte-derived chemokine (KC)-like protein in dogs as a biomarker of sepsis in dogs with bacterial uterine infection (pyometra). The aim was to compare KC-like concentrations in dogs with pyometra with or without sepsis and to assess associations between KC-like and clinical variables, including days of hospitalization as an outcome.

**Results:**

A mouse KC ELISA was validated and used to determine the concentrations of KC-like in serum from 34 dogs with pyometra and 18 healthy controls. Dogs with pyometra were classified as having sepsis based on two different criteria for systemic inflammatory response syndrome (SIRS), resulting in 74% and 30% sepsis-positive, respectively. The concentration of KC-like protein was higher in pyometra dogs with sepsis than in pyometra dogs without sepsis (*p* < 0.05) and in healthy controls (*p* < 0.0001) when using either of the two SIRS criteria. Moreover, KC-like was slightly increased in dogs with pyometra without sepsis compared with healthy controls when using the more stringent SIRS criteria (*p* < 0.05). Analyses of all dogs showed that KC-like concentrations correlated positively with hospitalization days, C-reactive protein (CRP) concentrations, white blood cells, and percentage of band neutrophils; however, KC-like correlated negatively with hemoglobin and did not correlate with circulating creatinine.

**Conclusions:**

Our results suggest that circulating KC-like protein increases in dogs with sepsis in pyometra and that KC-like is associated with more severe clinical illness. These findings support a potential role of KC-like as a biomarker of sepsis; however, the true identity of KC-like in dogs has yet to be uncovered.

**Supplementary Information:**

The online version contains supplementary material available at 10.1186/s12917-024-04271-w.

## Background

Sepsis is associated with significant morbidity and a leading cause of death in animals and humans worldwide. This syndrome is defined as life-threatening organ dysfunction caused by a dysregulated inflammatory response to infection [1]. Early identification and treatment of sepsis, including early administration of antimicrobials, are critical for survival [2, 3]. However, diagnosing sepsis can be challenging since the signs are often vague and unspecific. The management of sepsis is also demanding due to the complex pathophysiology with inflammatory mediators, cytokines, chemokines and acute phase reactants activated in overflow. The resulting cytokine storm triggers an ongoing reaction, leading to a dysregulated host response and organ dysfunction. In dogs, the mortality rates reported can be as high as 81.1% if septic shock develops [4]. The search for objective biomarkers that may help to diagnose sepsis early, for evaluating treatment response and for prognostication are thus highly warranted. The inflammatory responses in dogs and humans have many similarities, which is why biomarkers specific for dogs with sepsis may also be of interest for human medicine.

Pyometra is a potentially life-threatening hormone-induced purulent bacterial uterine infection that is common in female dogs. In some dog breeds, as many as 50% of all unspayed female dogs will develop the disease before 10 years of age, and standard clinical criteria for systemic inflammatory response syndrome (SIRS) [5] are fulfilled in the majority of cases [6–8]. In human medicine, instead of the SIRS criteria to clinically identify sepsis in patients with infection, a new definition including the application of Sequential Organ Failure Assessment (SOFA) scores or the simplified quick SOFA (qSOFA) scores is increasingly recognized [1, 9]. However, until validated corresponding new sepsis criteria for dogs are available, the currently validated SIRS criteria remain to be used for identifying sepsis in research studies [10–12]. However, there is a major risk of overdiagnosing SIRS (and sepsis in pyometra) due to the unspecific nature of these SIRS criteria; hence, more stringent criteria are needed for correct diagnosis and prognostication [13]. Dogs with pyometra have been studied as a model for spontaneously developed sepsis. Previous studies have identified several biomarkers that have diagnostic and/or prognostic potential, including acute phase proteins such as C-reactive protein (CRP) and serum amyloid A and several cytokines and chemokines [14–19].

Our previous study using a multiplex approach analyzed 12 cytokines and demonstrated that, compared to healthy controls, dogs with pyometra had higher concentrations of the keratinocyte-derived chemoattractant (KC)-like protein and that KC-like was associated with morbidity as measured by length of hospitalization [19]. Additionally, we have found indications for an increased KC-like concentration in septic dogs compared with nonseptic dogs with pyometra [19]. The potential value of KC-like as a biomarker was further supported by another study, in which concentrations of KC-like, C-X-C motif chemokine (CXCL)-8 and C-C motif chemokine ligand 2 (CCL2) were increased in septic dogs compared to healthy dogs, and CCL2 was independently associated with prognosis [20]. Moreover, increased KC-like concentrations have been detected in cats with sepsis or septic shock [21]. However, similar concentrations of KC-like in dogs with pyometra compared with healthy bitches in different stages of the estrous cycle were reported in another study [22]. Notably, detection of KC-like in dogs has thus far only been done by using the same multiplex assay, which frequently results in values below the detection limit [19, 20, 23], and to our knowledge, there are no reports using an independent single-analyte assay for validation of these KC-like measurements. Moreover, due to the very low sample size of dogs without sepsis in our previous study [19], the ability of KC-like to discriminate between septic and nonseptic dogs with pyometra is still elusive.

KC-like is a protein detected in canine blood samples that cross-reacts with antibodies against mouse KC, also known as CXCL1 and GRO alpha [24]. Mouse KC is a member of the CXC family of chemokines and is one of the major neutrophil chemoattractants in mice [25]. For instance, KC released by mast cells and macrophages leads to rapid recruitment of neutrophils in response to intraperitoneal administration of lipopolysaccharide (LPS) in mice [26]. It is feasible that KC-like protein in dogs exert similar functions as mouse KC and thus participate in the pathogenic accumulation of neutrophils in sepsis induced by bacterial infection of the uterus. It is, however, important to note that no KC-like gene has been identified thus far in the dog genome, and instead, we identified canine CXCL7, CXCL5, and CXCL8 as the closest homologs to mouse KC with total scores of 88.6%, 77.8%, and 75.5%, respectively [19]. Dog CXCL7 shares 46% sequence identity with the mouse KC protein and has been shown to be a potent chemoattractant and neutrophil activator [27], hence representing an attractive candidate for the KC-like protein in dogs.

In the present study, we aimed to further study the potential of KC-like as a biomarker of canine sepsis using pyometra as a model for naturally developing sepsis. We have validated and used a murine KC ELISA for measuring KC-like concentrations in sera from dogs with pyometra, with or without sepsis according to two different SIRS criteria, and in healthy control dogs. We also evaluated correlations between KC-like and sepsis-related clinical and hematological variables. We hypothesized that KC-like concentrations increase in septic dogs and correlate with disease severity in pyometra.

## Results

### Sepsis classification and clinical characteristics of dogs

For comparison, we used two different groups of SIRS criteria based on clinical parameters to classify dogs with pyometra as either septic or nonseptic. The highest number of SIRS-positive dogs was obtained with the conventional criteria validated by Hauptman et al. [5], resulting in 74% septic dogs (25 of 34 dogs with pyometra). In contrast, the more stringent SIRS criteria described by Okano et al. [13] resulted in only 30% septic dogs (10 of 33 pyometra cases, with one dog excluded due to incomplete data required for classification.

The clinical and laboratory variables of septic and nonseptic dogs with pyometra according to conventional criteria [5] and healthy controls are summarized in Table 1. There was no difference in age or weight between septic and nonseptic dogs with pyometra. However, the age of healthy control dogs was lower than that of dogs with sepsis and pyometra (*p* ≤ 0.01). Compared with healthy dogs, hemoglobin concentrations were higher in both septic and nonseptic dogs with pyometra (*p* ≤ 0.001), whereas creatinine and CRP concentrations were higher only in the septic dogs with pyometra (*p* ≤ 0.05 and *p* ≤ 0.001, respectively). Hemoglobin, creatinine or CRP concentrations did not differ between septic and nonseptic dogs with pyometra.

Antimicrobials were used in two dogs with pyometra, both classified as septic dogs according to the conventional criteria and nonseptic dogs according to the more stringent criteria. One was treated with aminopenicillin (ampicillin) for 11 days prior to inclusion in the study, and the other was treated with the same drug but with no antimicrobials administered two days prior to inclusion. In both dogs, *E. coli* was isolated from the uterine (abundant growth) and blood cultures.

**Table 1:** Clinical and laboratory variables of dogs^a^

	Pyometra septic	Pyometra nonseptic	Healthy controls
	(*n* = 25)^b^	(*n* = 9)^b^	(*n* = 18)
Age (years)	7.2 (5.9-9.0)**	6.5 (2.6–9.05)	4.2 (2.78–6.25)
Weight (kg)	25.3 (18.1–34.3)	30.0 (16.5–32.8)	23.0 (13.68–30.25)
Hospitalization (days)	2 (2–3)	2 (2–3)	NA
Temperature (°C)	39.0 (38.25–39.4)**	38.8 (38.6-38.95)*	38.25 (38.1-38.33)
HR (min^− 1^)	100 (89–120)	100 (86.5–105)	92 (80–114)
RR (min^− 1^)	29 (24–57)*(**)^c^	16 (16–20)	20 (16–30)
WBC (x10^9^ L^− 1^)	21.0 (16.3-28.75)***(*)	11.5 (8.3–17.6)	8.65 (7.65-10)
PBN (%)	19.5 (12.75–43.6)***(*)	3.8 (0.3–15)^d^	1.8 (0.0-11.35)
Hb (g L^− 1^)	129 (120-151.5)***	134 (117–145)***	164 (151–177)
Creatinine (µmol L^− 1^)	59 (49-66.5)*	59 (55.5–65.5)	75 (61-80.25)
CRP (mg L^− 1^)	206 (126.5-281.5)***	7.0 (5.0-212)	5.0 (5.0-5.75)^e^

^a^ Variables are expressed as median (interquartile range). NA, not applicable (only 3 dogs were operated on, all with hospitalization for 2 days). Pyometra septic: pyometra and ≥ 2 criteria fulfilled for SIRS [5]; pyometra nonseptic: pyometra and fulfilling < 2 criteria for SIRS; HR: heart rate; RR: respiratory rate; WBC: total white blood cell count; PBN: percentage of band neutrophils; Hb: hemoglobin; CRP: C-reactive protein.

^b^ *p < 0.05, **p < 0.01, ***p < 0.001, compared to the control group; (*)*p* < 0.05, (**)*p* < 0.01, (***)*p* < 0.001, compared to the nonseptic pyometra group.

^c^ Three missing values due to imprecise data.

^d^ One missing value.

^e^ Two missing values.

### Higher serum concentration of KC-like in septic dogs with pyometra

**Fig. 1:**
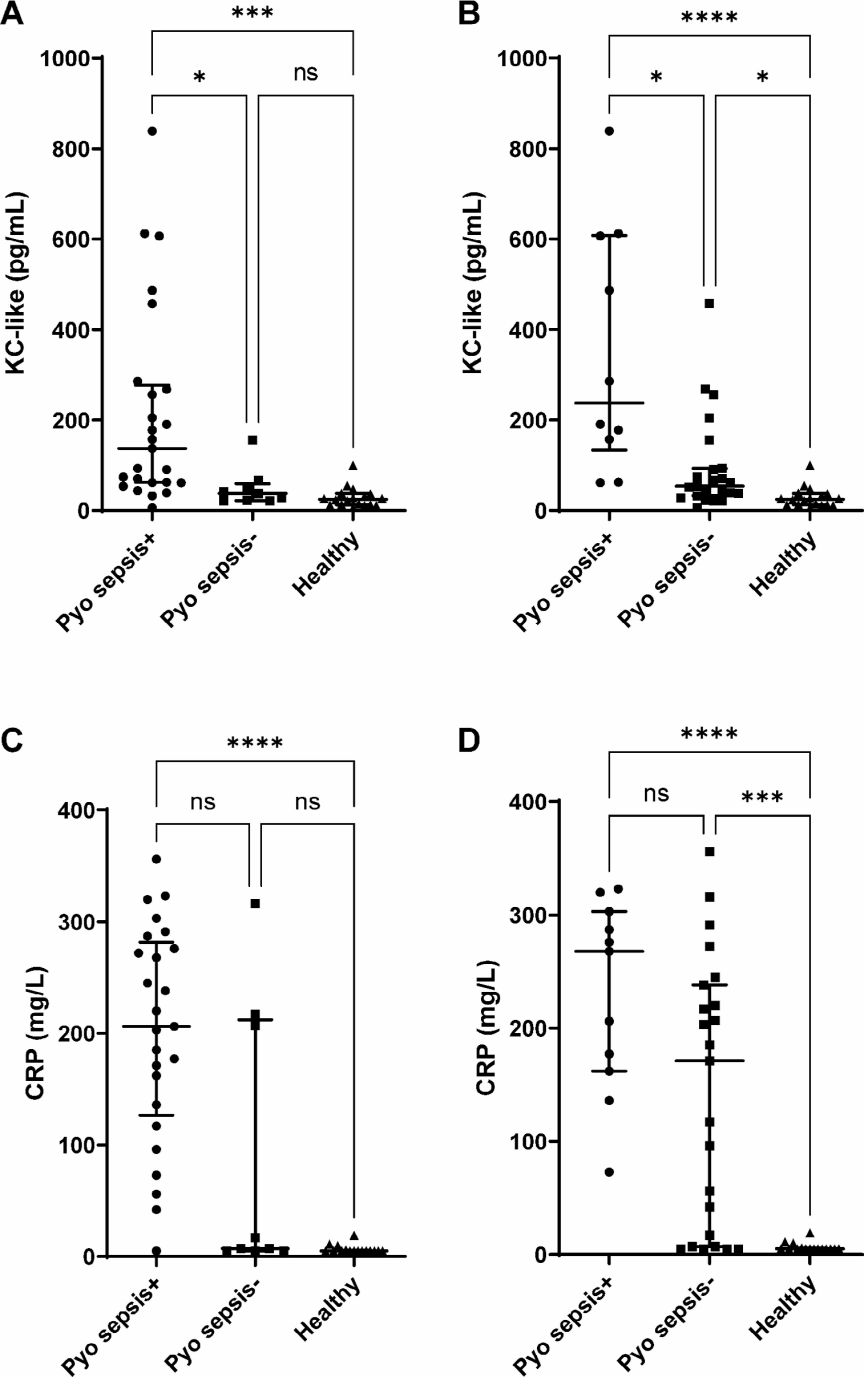
Increased KC-like and CRP concentrations in dogs with pyometra and sepsis. KC-like was measured with ELISA and CRP was measured with two automated methods in serum from dogs with pyometra and sepsis (pyo sepsis+, *n* = 25 in **A** and **C**, *n* = 10 in **B** and **D**), dogs with pyometra without sepsis (Pyo sepsis-, *n* = 9 in **A** and **C**, *n* = 23 in **B** and **D**) and healthy controls (Healthy, *n* = 18). Sepsis classifications were based on the conventional [5] (**A** and **C**) or the alternative [13] (**B** and **D**) SIRS criteria as described in the methods. Data are presented as individual values in addition to medians and interquartile ranges. Differences between groups are indicated as follows: *p < 0.05, ***p < 0.001, ****p < 0.0001, and ns (not significant) *p* > 0.05

To determine whether concentrations of KC-like could discriminate between septic and nonseptic dogs with pyometra and healthy controls, we measured KC-like protein in serum from the three groups of dogs using a mouse KC ELISA that has been validated as described in the Methods section. KC-like concentrations in all dogs with pyometra (including septic and nonseptic dogs) did not differ statistically between the first sample collection in 2011–2013 (137 pg/mL [43.5–268], *n* = 23) and the second collection in 2017 (61.5 pg/mL [37.7–90.7], *n* = 11, *p* = 0.176). KC-like concentrations were higher in dogs with pyometra and sepsis (137 pg/mL [61.9–277]) than in dogs with pyometra and no sepsis (37.7 pg/mL [22.0–59.4], *p* < 0.05) and healthy controls (24.6 pg/mL [12.3–37.4], *p* < 0.0001) when using the conventional SIRS criteria for classifications [5] (Fig. 1A). There was no statistically significant difference in KC-like concentrations between nonseptic dogs with pyometra and healthy controls (Fig. 1A).

With the alternative and more stringent SIRS criteria [13], resulting in a reduced septic group and increased nonseptic group, there was still a higher KC-like concentration in septic dogs with pyometra (238 pg/mL [133–698], *p* < 0.05) than in nonseptic dogs with pyometra (53.7 pg/mL [32.5–93.4], *p* < 0.0001) and healthy controls (24.6 pg/mL [12.3–37.4], pg/mL, *p* < 0.0001)(Fig. 1B). With stricter sepsis classification, the concentrations of KC-like in nonseptic dogs with pyometra were also higher than those in healthy controls (*p* < 0.05). In a separate analysis using log-transformed data, KC-like concentrations differed significantly between the same groups of dogs, as shown in Fig. 1 (Supplementary Fig. 1, Additional File 1). The serum concentration of CRP was increased in septic dogs with pyometra compared to controls but did not differ between septic and nonseptic dogs with pyometra, regardless of which SIRS criteria was used (Fig. 1C and D).

### Correlations between serum KC-like and clinical features

**Fig. 2:**
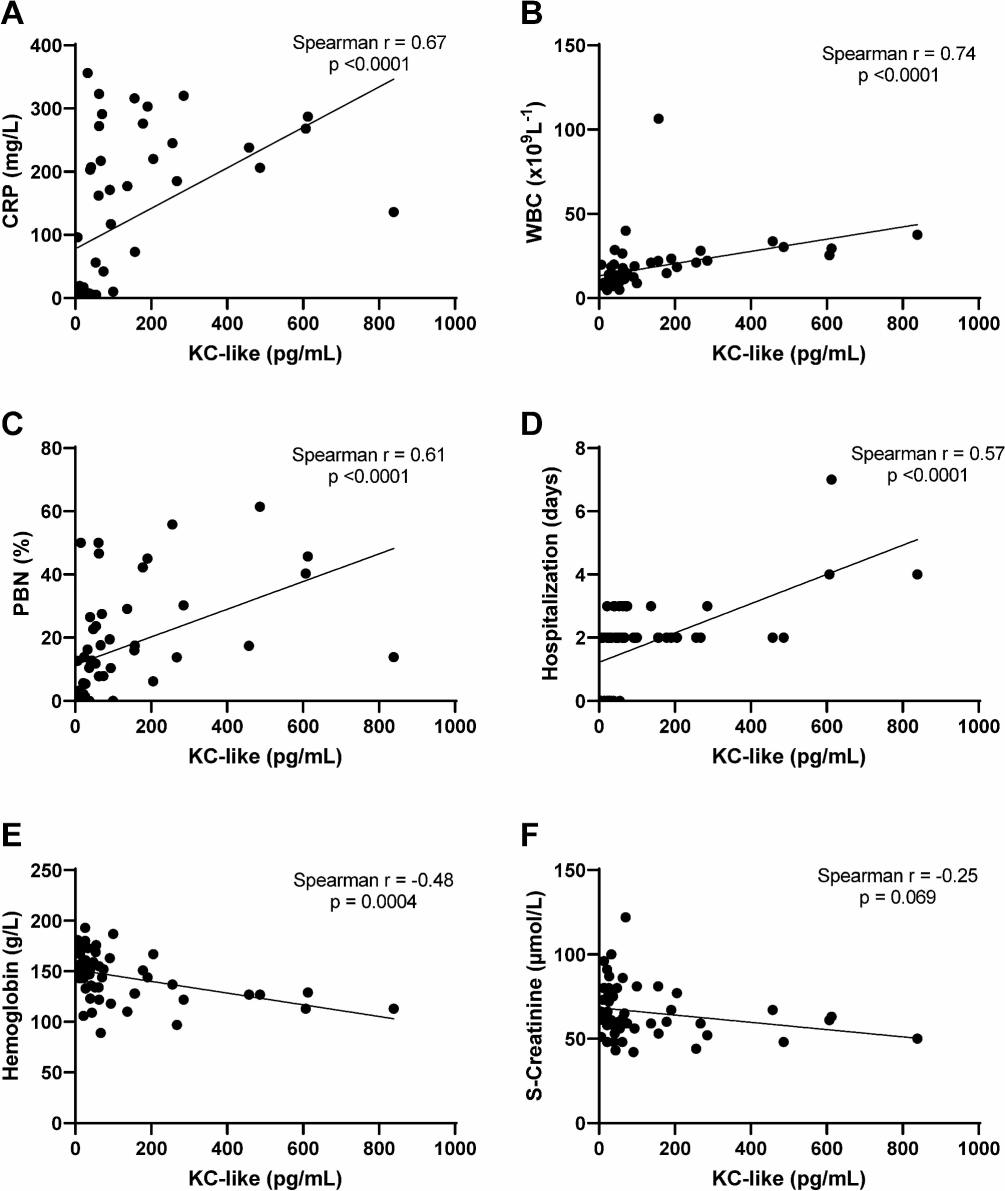
Correlations between KC-like concentrations and clinical and hematological parameters. KC-like correlated positively with C-reactive protein (CRP), white blood cell count (WBC), percent band neutrophils (PBN), and days of hospitalization and negatively with hemoglobin. There was no correlation between KC-like and serum creatinine (S-creatinine). Analyses were performed with Spearman’s rank correlation coefficients and included all dogs in the study (*n* = 52)

Strong and positive correlations were found between concentrations of KC-like and concentrations of CRP, white blood cell counts (WBC) and percentage band neutrophils (PBN) (Fig. 2A-C). Moreover, KC-like concentrations were moderately and positively correlated with days of hospitalization and moderately and negatively correlated with hemoglobin concentrations, while no correlation was observed with S-creatinine (Fig. 2D-F). Both CRP and PBN demonstrated positive correlations with length of hospitalization (Supplementary Fig. 2, Additional File 2).

## Discussion

In this study, we demonstrated that the KC-like concentration in canine serum, as measured by a validated murine KC ELISA, could discriminate between septic and nonseptic dogs with pyometra using two different SIRS criteria for sepsis classification. We also found that KC-like correlated positively with days of hospitalization, serum CRP, WBC and PBN, but negatively with hemoglobin. Similar to KC-like, the conventional biomarker CRP correlated positively with length of hospitalization, which has been shown previously [7], however, CRP did not discriminate between septic and nonseptic dogs with pyometra in this study. Together, these findings confirm our previous study indicating a role for KC-like in canine sepsis related to pyometra [19] and they suggest that KC-like has the potential as a biomarker to distinguish septic from nonseptic dogs with pyometra.

Although the epitope of the mouse KC antibodies used in multiplex or ELISA analyses is not known, we may speculate that the closest homolog CXCL7 alone, or together with the other two related chemokines CXCL5 and CXCL8 [19], contributed to the detection of the KC-like protein in dog serum. However, the true identity of the KC-like protein in dogs remains unknown, which may raise questions about the specificity of this biomarker. Hence, future studies to reveal the nature of KC-like are warranted.

A major finding of this study was the increased expression of KC-like in dogs with sepsis related to pyometra. Studies from other groups have also measured KC-like in dogs with sepsis and/or pyometra, however, none of those studies has assessed the difference between septic and nonseptic dogs with pyometra. Our previous study demonstrated an increased KC-like concentration in dogs with pyometra, regardless of sepsis or not, compared with healthy dogs [19]. Another study, however, reported no difference in KC-like concentrations between dogs with pyometra and healthy dogs in anoestrus, dioestrus or pregnant states [22]. This discrepancy could be due to the differences in measurement methods, pyometra severity or demographics of healthy dogs. In agreement with the present study, higher KC-like concentrations have been found in sepsis cases compared with healthy controls both in dogs [20, 28] and in cats [21].

Pyometra has been shown to induce endotoxemia in dogs [16], and administration of *Escherichia coli* LPS to healthy dogs could transiently induce the expression of KC-like and several other cytokines in an experimental model of sepsis [29]. Moreover, KC-like was one of several biomarkers that was decreased upon antimicrobial drug treatment in dogs with pneumonia, septic peritonitis or pyometra [30]. Together, these studies implicate KC-like in the inflammatory process caused by bacterial infection. However, in a study of several potential biomarkers in dogs, KC-like did not discriminate between septic peritonitis and nonseptic ascites, as did blood concentrations of CCL2 and peritoneal effusion concentrations of CCL2, IL-6, IL-10 and lactate [10]. This suggests that KC-like may not be a general biomarker for sepsis in all types of canine bacterial infections, and CCL2 or other blood biomarkers may also have a role in discriminating septic from nonseptic cases in pyometra.

Notably, increased KC-like concentrations have also been shown in inflammatory conditions other than pyometra and sepsis, including noninfectious SIRS [20], immune-mediated hemolytic anemia [28, 31], babesiosis [32, 33], diabetes mellitus [34] and obesity [35]. Together, these findings demonstrate that KC-like expression is not restricted to bacterial infections or sepsis but can be induced by many different conditions, which may limit the value of KC-like as a single sepsis biomarker in clinical practice. However, KC-like may still be useful in combination with other biomarkers to predict the development of complications in inflammatory settings, as was suggested by a study of dogs infected with the tick-borne parasite *Babesia canis* [33]. For instance, it may be beneficial to use KC-like in combination with CRP, which is a well-known diagnostic marker for systemic inflammation in various disorders [36].

Although a majority of dogs with the uterine infection pyometra will fulfil the currently used clinical criteria for sepsis, a minority of these will develop the more acute and life-threatening septic shock. Dogs in the present study were included in the sepsis group (pyometra and SIRS) based on previously validated criteria that have been frequently used in research [5]. However, these criteria have a high sensitivity but are relatively nonspecific since temperature and heart and respiratory rates may be affected by many other causes, such as pain, stress, or fear. Additionally, normal dogs can have respiratory rates up to 30 breaths per minute, which exceeds the ≥ 20 breaths per minute used to fulfil the score for respiratory rate [37]. To avoid the inclusion of nonseptic dogs in the septic group, we also used the stricter criteria developed by Okano et al. [13], although these criteria have not been fully validated. With the conventional criteria we obtained a relatively low number of nonseptic dogs (*n* = 9), hence reducing the statistical power of the analyses. However, the comparable results obtained with both criteria may compensate for this weakness to some extent.

One limitation of this study was the younger age in the group of healthy dogs than in the dogs with pyometra and sepsis. Although similar KC-like concentrations have been reported in young and old dogs, including both sexes [38], one study has demonstrated that the KC-like concentration in female dogs increases with higher age [39]. It is therefore possible that the difference in age in the present study may skew the results comparing KC-like between healthy controls and dogs with pyometra and sepsis. However, the most important finding in this study was that KC-like concentrations could discriminate septic from nonseptic dogs with pyometra and there was no age difference between these two groups of dogs; hence, age difference between the healthy control group and the pyometra groups may not affect the conclusion of this study.

Another limitation in this study was the long time span between the two sampling periods (one in 2011–2013 and another in 2017), leading to different storage times of samples before analysis, which could affect cytokine measurement. We cannot exclude the possibility that this has influenced our data, although there was no difference in concentration between sampling periods when analyzing serum from all dogs with pyometra. The potential negative effects of varying storage times could at least to some extent be reduced by the fact that dogs from both sampling periods were included in all study groups. The long-term stability of KC-like in frozen serum is, to our knowledge, not known, but we found no effect of up to three freeze-thaw cycles on the measured KC-like concentration with the KC ELISA kit, suggesting robust short-term stability.

## Conclusion

Here, we demonstrated that serum concentrations of KC-like differed between septic and nonseptic dogs with pyometra and that KC-like correlated with several clinical and hematological features of pyometra, including days of hospitalization. Our findings support a role for KC-like as a biomarker of sepsis and disease severity in pyometra. However, future studies are warranted to further evaluate the usefulness of KC-like as a predictive biomarker of outcome in pyometra, either alone or in combination with other biomarkers. Moreover, further investigations are necessary to identify the true nature of KC-like in dogs.

## Materials and methods

### Dogs and study design

Serum samples from 34 dogs with pyometra and 18 healthy controls, from 24 different dog breeds, were included in this study. The diagnosis of pyometra was based on history, physical examination, laboratory analyses including postoperative bacteriological culture of the uterine content and macroscopic and histopathological examination of the uterus and ovaries [19]. Dogs with concurrent diseases and dogs that had received anti-inflammatory medications prior to sampling were excluded. The selection of healthy controls was based on history, physical examination, laboratory variables and histopathological examination of the uterus and ovaries. None of the included healthy control dogs were treated with antimicrobials or other drugs. Written informed consents were obtained from all dog owners. The study was approved by the local ethical committee with permission numbers C242/7 and 21/2016. All methods used were in compliance with the EU Directive 2010/63/EU.

Dogs with pyometra were further divided into septic and non-septic groups according to the conventional SIRS criteria [5]. Using these SIRS criteria, the presence of sepsis e.g. the septic group (*n* = 25) was defined as pyometra and fulfilment of two or more of the following four clinical parameters: temperature < 38.1 °C or > 39,2 °C), tachycardia (> 120 beats per minute), tachypnoea (respiratory rate > 20 breaths per minute), and white blood cell count < 6 or > 16 × 10^9^ cells/L or left shift (> 3% band neutrophils). Dogs with pyometra that did not meet the SIRS criteria were assigned as the non-septic group (*n* = 9).

For comparison, we also used the stricter SIRS criteria presented by Okano et al. [13] in our analysis by which sepsis was assigned to dogs with pyometra that fulfilled two or more of the following clinical parameters: temperature < 37.8 °C > 39,7 °C), tachycardia (> 160 beats per minute), tachypnea (respiratory rate > 40 breaths per minute), and white blood cell count < 4 × 10^9^ or > 12 × 10^9^ cells/L or left shift (> 10% band neutrophils). This resulted in the classification of 10 dogs with sepsis and 23 dogs without sepsis. One dog was excluded from this analysis due to missing exact information for respiratory rate (tachypnea > 30 breaths per minute).

### Clinical and laboratory variables

The physical examination of all dogs were performed at the University Animal Hospital, Swedish University of Agricultural Sciences, Uppsala, Sweden including assessments of body weight, body temperature, heart rate, respiratory rate, hydration status, mucus membrane color and appearance, abdominal palpation, general condition, lymph nodes, capillary refill time and vaginal palpation. Standard hematological and biochemical analysis for white blood cell count (WBC), differential blood cell count, hemoglobin concentration and packed cell volume (Advia 2120, Siemens Healthcare Diagnostics, Deerfield, IL. USA) were performed at the Clinical Pathology Laboratory, University Animal Hospital, Swedish University of Agricultural Sciences. The serum concentration of C-reactive protein (CRP) was measured with an automated assay (High Linearity CRP, Randox Laboratories, Crumlin, United Kingdom) [40, 41] or an automated immunoturbidimetric method (Gentian cCRP, Gentian AS, Moss, Norway) validated for dog serum, yielding similar results as the Randox method [42]. The serum concentration of creatinine was measured using the Abbott Architect c4000 chemistry analyzer (Abbot Park, IL, USA).

### Serum sample preparation

At the time of admission and prior to treatment, venous blood was collected at the University Animal Hospital, Swedish University of Agricultural Sciences, Uppsala, Sweden. Serum was prepared by a standardized protocol and stored in aliquots at -80 °C until further analysis.

### KC-like measurement by ELISA

KC-like protein concentration in dog serum was measured by using a mouse CXCL1 (KC) ELISA kit (EMCXCL1, Thermo Fisher Scientific, Waltham, MA, USA) that was validated for dog serum (see below for validation process). This kit was selected based on our earlier study of this cytokine included in a canine multiplex immunoassay [23]. For measurement of KC-like in dog serum, samples were diluted 5-fold and analyzed in duplicate or triplicate in accordance with the instructions from the manufacturer within 30 min by measuring absorbance at 450 nm using the Tecan Sunrise microplate reader (Tecan Group Ltd., Männedorf, Switzerland).

In addition to the mouse KC ELISA kit, we also evaluated potential cross-reactivity with dog CXCL-5 using a human ENA-78 ELISA kit that measures human CXCL-5 (ELH-ENA78, RayBiotech, Norcross, GA, USA). However, values above detection limit were not obtained in 21 analyzed serum samples from dogs with pyometra, of which 13 had sepsis, and therefore, we did not use this kit further since we could not confirm any cross-reactivity with CXCL-5 in dog serum.

### Validation of the mouse KC ELISA for detection of dog KC-like in serum

The mouse CXCL1 (KC) ELISA kit (EMCXCL1, Thermo Fisher Scientific) was validated for dog serum. All samples and calibrators were diluted with the assay diluent provided in the ELISA kit. Experimental samples were diluted 5-fold and analyzed in triplicate. The intra-assay coefficient of variation (CV) based on triplicates of five dog serum samples was 4.5+/-2.5% (mean+/-SD). To determine inter-assay precision, two serum samples with low- or medium-level of KC-like (34.0 and 53.0 pg/ml) were analyzed in triplicate in two independent runs, resulting in an inter-assay CV% of 8.3+/-3.1% (mean+/-SD). For assessment of the linearity of dilution, a high-level serum sample containing 268 pg/ml KC-like was used. The recoveries of sample dilutions at 1:1 (neat), 1:2, 1:4, 1:8 and 1:16 ratios were 48.2, 81.4, 100, 105 and 106%, respectively, when using the 1:4 dilution as the reference. Spike and recovery were determined by adding 75 pg/ml recombinant mouse CXCL1 standard (calibrator) to three low- or medium-level samples (21.6, 32.1 and 94.7 pg/ml) diluted 1:5, resulting in a recovery of 58.4 +/-7.4% (mean+/-SD). The recoveries of spiked samples diluted 1:2, 1:4 and 1:8 were 114, 127, and 126%, respectively. To determine sample stability, a medium-level serum sample (52,3 pg/ml) was subjected to one, two or three freeze-thaw cycles, resulting in a recovery of 102+/-3.7% (mean+/-SD) compared with the unthawed sample.

### Statistical analysis

Statistical analyses were performed with GraphPad Prism 9.0 (GraphPad Software Inc., San Diego, CA, USA). Most variables were not normally distributed according to the Kolmogorov‒Smirnov normality test; hence, the data are presented as medians and interquartile ranges. Differences between groups were analyzed by the Kruskal‒Wallis test with Dunn’s multiple comparisons test. Correlations were determined by Spearman’s rank correlation coefficients. Strengths of correlations were valued as very weak (*r* < 0.2), weak (*r* = 0.2 to 0.39), moderate (*r* = 0.4 to 0.59), strong (*r* = 0.6 to 0.79), or very strong (*r* = 0.8–1). A value of *p* < 0.05 was considered significant. In a separate analysis, log transformation of raw data was performed for KC-like protein to compensate for non-normality as determined by the Kolmogorov‒Smirnov normality test, and differences between groups were analyzed by one-way ANOVA and Bonferroni’s multiple comparisons test.

## Electronic supplementary material

Below is the link to the electronic supplementary material.

Additional file 1: supplementary Fig. 1: Analysis of log-transformed data demonstrates increased KC-like concentrations in dogs with pyometra and sepsis

Additional file 2: supplementary Fig. 2: Positive correlations between CRP or PBN and length of hospitalization

## Data Availability

The datasets used and/or analyzed during the current study are available from the corresponding author on reasonable request.

## Abbreviations

CCL: Chemokine (C-C motif) ligand
CRP: C-reactive protein
CV: Coefficient of variation
CXCL: Chemokine (C-X-C motif) ligand
ELISA: Enzyme-linked immunosorbent assay
KC: Keratinocyte-derived chemokine
LPS: Lipopolysaccharide
SD: Standard deviation
SIRS: Systemic inflammatory response syndrome
PBN: Percentage band neutrophils
WBC: White blood cell counts
