# Supplementary material for: KC-like chemokine as a biomarker of sepsis in dogs with pyometra
Source: BMC Vet Res. 2024 Sep 13;20:411. doi: 10.1186/s12917-024-04271-w (PMC11395178; doi:10.1186/s12917-024-04271-w)
Supplement: Supplementary file 1 — Additional file 1: supplementary Fig. 1: Analysis of log-transformed data demonstrates increased KC-like concentrations in dogs with pyometra and sepsis [file 12917_2024_4271_MOESM1_ESM.pdf]

Supplementary Fig. 1

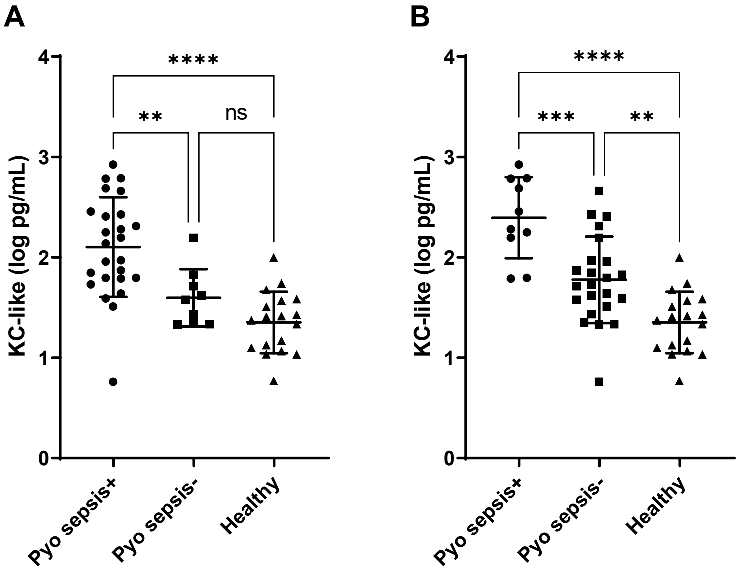

Supplementary Figure 1. Analysis of log-transformed data demonstrates increased KC-like concentrations in dogs with pyometra and sepsis. KC-like was measured with ELISA in dogs with pyometra and sepsis (pyo sepsis+, n=25 in A, n=10 in B), dogs with pyometra without sepsis (Pyo sepsis-, n=9 in A, n=23 in B) and healthy controls (Healthy, n=18). Sepsis classifications were based on the conventional (A) or the alternative (B) SIRS criteria as described in the methods. Log-transformed data is presented as individual values in addition to means and standard deviations. Differences between groups are indicated as follows: **p<0.01, ***p<0.001, ***p<0.0001, and ns (not significant) p>0.05.
