# Supplementary material for: KC-like chemokine as a biomarker of sepsis in dogs with pyometra
Source: BMC Vet Res. 2024 Sep 13;20:411. doi: 10.1186/s12917-024-04271-w (PMC11395178; doi:10.1186/s12917-024-04271-w)
Supplement: Supplementary file 2 — Additional file 2: supplementary Fig. 2: Positive correlations between CRP or PBN and length of hospitalization [file 12917_2024_4271_MOESM2_ESM.pdf]

Supplementary Fig. 2

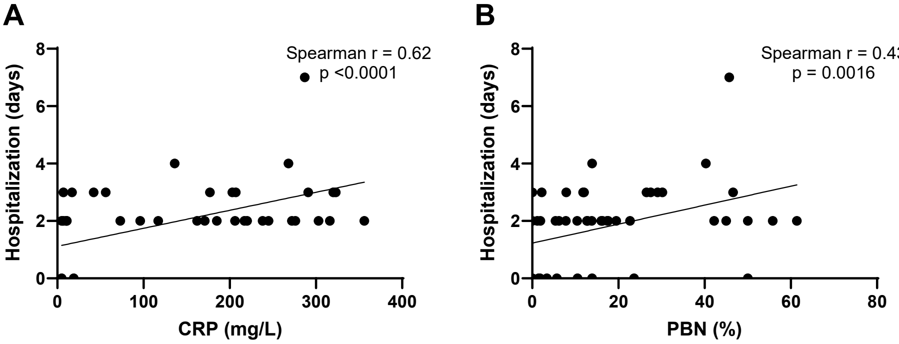

Supplementary Figure 2. Positive correlations between CRP or PBN and length of hospitalization. Analyses were performed with Spearman's rank correlation coefficients and included all dogs in the study (n=52).
